# Promoting Wound Healing Using Low Molecular Weight Fucoidan in a Full-Thickness Dermal Excision Rat Model

**DOI:** 10.3390/md15040112

**Published:** 2017-04-07

**Authors:** Jun-Hyeong Park, Seong-Hun Choi, Soo-Jin Park, Young Joon Lee, Jong Hyun Park, Phil Hyun Song, Chang-Mo Cho, Sae-Kwang Ku, Chang-Hyun Song

**Affiliations:** 1 Department of Anatomy and Histology, College of Korean Medicine, Daegu Haany University, Gyeongsan 38610, Korea; kjadjh123@naver.com (J.-H.P.); ck0190@hanmail.net (S.-H.C.); sjp124@gmail.com (S.-J.P.); 2 Department of Preventive Medicine, College of Korean Medicine, Daegu Haany University, Gyeongsan 38610, Korea; gksxntk@dhu.ac.kr; 3 Department of Pathology, College of Korean Medicine, Daegu Haany University, Gyeongsan 38610, Korea; moguri@dhu.ac.kr; 4 Department of Urology, College of Medicine, Yeungnam University, Daegu 42415, Korea; sph04@yu.ac.kr; 5 Faculty of Physical Education, College of Physical Education, Keimyung University, Daegu 42601, Korea

**Keywords:** skin, wound, low molecular weight, fucoidan, antioxidant, anti-inflammation

## Abstract

Low molecular weight fucoidan (LMF) has been reported to possess anti-inflammatory and antioxidant activities. Thus, we examined the effects of LMF extracted from *Undaria pinnatifida* on dermal wounds. Five round dermal wounds were created on the dorsal back of rats, and they were then treated topically with distilled water (DW), Madecasol Care™ (MC) or LMF at 200, 100 and 50 mg/mL, twice a day for a week. There were dose-dependent increases in wound contraction in the groups receiving LMF but not in the MC group, compared with the DW. Histopathological examination revealed that LMF treatment accelerated wound healing, which was supported by increases in granular tissue formation on day four post-treatment but a decrease on day seven, accompanied by an evident reduction in inflammatory cells. In the LMF-treated wounds, collagen distribution and angiogenesis were increased in the granular tissue on days four and seven post-treatment. Immunoreactive cells for transforming growth factor-β1, vascular endothelial growth factor receptor-2 or matrix metalloproteinases 9 were also increased, probably due to tissue remodeling. Furthermore, LMF treatment reduced lipid peroxidation and increased antioxidant activities. These suggested that LMF promotes dermal wound healing via complex and coordinated antioxidant, anti-inflammatory and growth factor-dependent activities.

## 1. Introduction

Dermal wound healing is a physiological process that restores the anatomical structure and function of injured skin. Wound healing proceeds via three overlapping phases: inflammatory phase with hemostasis and inflammation; proliferative phase with angiogenesis, collagen deposition, granulation tissue formation and epithelialization; and remodeling phase with connective tissue deposition [1]. It involves the interaction of a complex cascade of various cells including leukocytes, blood cells, fibroblasts, and epithelial cells [2]. However, extended secretion of pro-inflammatory cytokines, such as tumor necrosis factor-α (TNF-α) or interleukin (IL)-1, prolongs the inflammatory phase resulting in chronic wounds or hypertrophic scar formation [3]. Furthermore, several cytokines as inflammatory mediators and the resultant free radicals increase reactive oxygen species (ROS) levels and enzyme-induced tissue damage, and decrease endogenous antioxidants, which also results in impaired wound healing [4]. 

Most conventional treatments for dermal wounds, such as nonsteroidal anti-inflammatory drugs (NSAIDs) and topical corticosteroids, aim to reduce inflammation [5]. However, the treatment can have a negative impact on wound healing, including adverse effects such as atrophy, osteoporosis, obesity and glaucoma [6]. For complementary and alternative medicine, many natural sources rich in various nutritional factors, including proteins, carbohydrates and vitamins, have been attempted to reveal the promotion of wound healing, because restoring injured dermal tissues requires an amount of energy [1]. Furthermore, their antioxidant activities have shown therapeutic potential of these resources for wound healing [7,8,9,10]. The major components of natural extracts such as flavonoids, phenols and tannins are known to act as free radical scavengers [11]. This raises the intriguing possibility of using natural resources for dermal wound healing, but the scientific evidence for the effects of potential bioactive compounds and their therapeutic benefits is lacking [12].

Fucoidans are a family of sulfated polyfucose polysaccharides extracted from brown marine algae [13]. They have received significant biotechnological research interest because of their antitumor [14], immunomodulatory [15], antioxidant [16], antivirus [17], anticoagulant [18] and anti-inflammatory [19] effects as well as for being an abundant non-animal origin and non-toxic edible resource [16]. Fucoidans possibly interact with a variety of growth factors such as basic fibroblast growth factor (bFGF) [20] and transforming growth factor-β [21], through mechanisms similar to that of heparin binding, to mediate these beneficial effects. However, since the high molecular weight fucoidans (HMF) are difficult to absorb and thus have low bioavailability in tissues, reducing the molecular weight of fucoidans is considered a reasonable way to enhance their bioactivity [22]. The therapeutic potential of low molecular weight fucoidan (LMF) has been revealed in animal models of arteriosclerosis [23], artery stenosis [24] and hindlimb ischemia [25]. The beneficial effects of LMF on anti-inflammation and angiogenesis suggest their clinical potential for dermal wound healing, but there have been no specific pharmaceutical or dermatological applications using LMF. Our previous study demonstrated favorable pharmacological and toxicological profiles of LMF extracted from the sporophylls of *Undaria pinnatifida* (known as Wakame, an edible seaweed) [26,27], which is a part of the food culture in Asia, particularly in Korea, Japan and the Philippines, and has high amounts of sulfate and l-fucose compared with other sulfated polysaccharides [28]. The therapeutic effects of LMF were associated with its strong antioxidant properties. Thus, the present study aimed to determine whether LMF promotes wound healing in a full-thickness dermal excision rat model in comparison with Madecassol Care™ (MC) containing 1% *Centella asiatica* as a commercial natural source [29].

## 2. Results

### 2.1. Promotion of Wound Contraction

**Figure 1:**
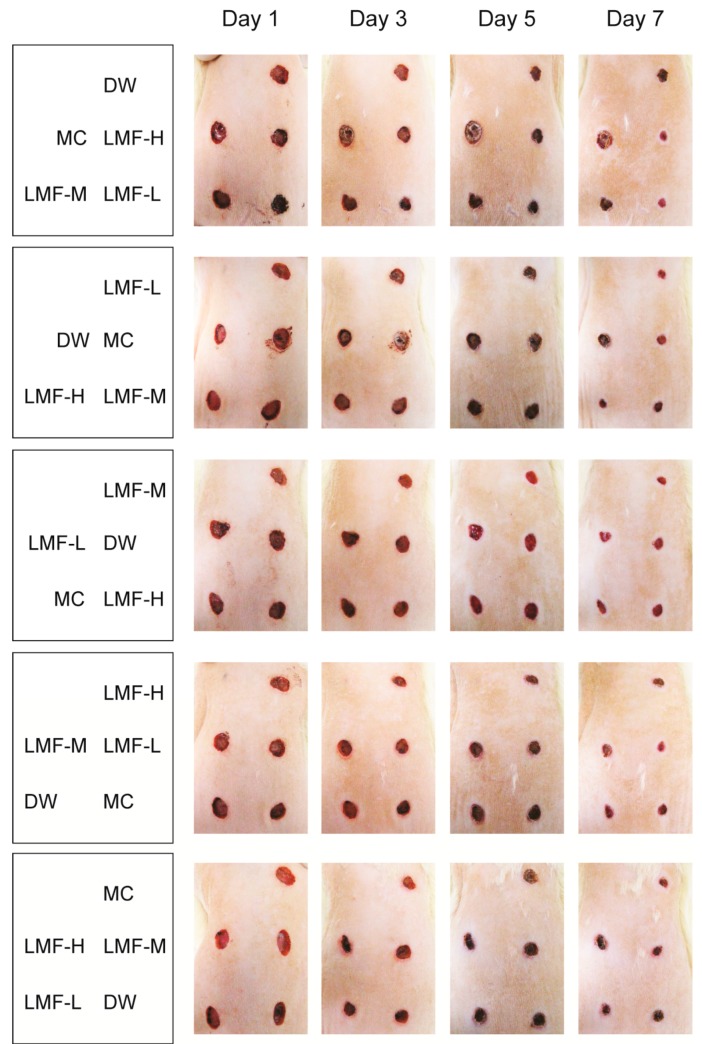
Gross aspects of dermal wound healing. Five round wounds were made in the dorsal backs of rats, and the wounds were treated twice a day for a week as indicated in the first column. The representative photographs were taken on days 1, 3, 5, and 7 post-treatment.

**Figure 2:**
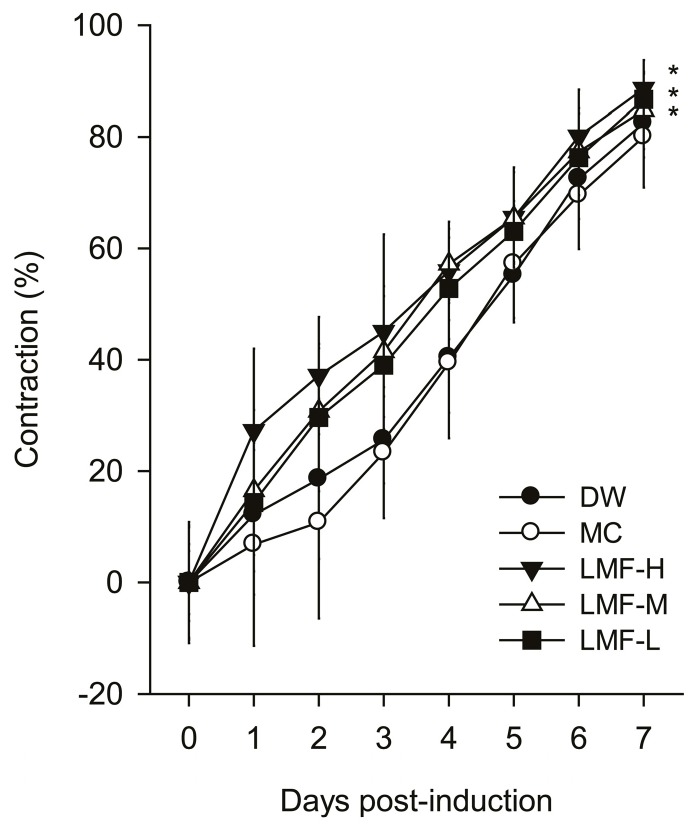
Dermal wound contraction. The area of each dermal wound was assessed daily for a week, and expressed as a percentage of reduced wound area to the area of the initial wound. Values are represented as means ± SD for 10 wounds per treatment. An asterisk indicates significance at *p* < 0.05.

The excised dermal wounds exhibited similar areas (37.9–39.4 mm^2^) prior to treatment, however the progress of wound healing was different depending on the treatment group and wound location (Figure 1). Three-way ANOVA for the kinetics of wound contraction showed significant main effects for group (*F* = 9.8; *p* < 0.01), region (*F* = 5.6; *p* < 0.01), and day (*F* = 613.9; *p* < 0.01) (Figure 2). It also showed significant interactions between group and day (*F* = 2.3; *p* < 0.01), but no interactions between group and region. The post-hoc tests versus distilled water treated group (DW) revealed significant increases in the contraction ratio of the three groups treated with LMF (*p* < 0.01), but not in that of the MC group. The increases were detected on days 1–7 post-treatment in LMF-H (LMF at 200 mg/mL) and days 2~5 in LMF-M (LMF at 100 mg/mL) and LMF-L (LMF at 50 mg/mL) (*p* < 0.05). The half-closure time (CT_50_) was significantly decreased only in the groups treated with LMF, compared with that in the DW and MC groups (*p* < 0.05). The CT_50_ was days 3.2 ± 1.2, 3.5 ± 0.6 and 3.8 ± 0.9 post-treatment in the groups of LMF-H, LMF-M and LMF-L, respectively, while it was days 4.6 ± 0.5 and 4.6 ± 0.6 in groups of DW and MC, respectively.

### 2.2. Wound Healing Effects on Day 4 Post-Treatment

**Figure 3:**
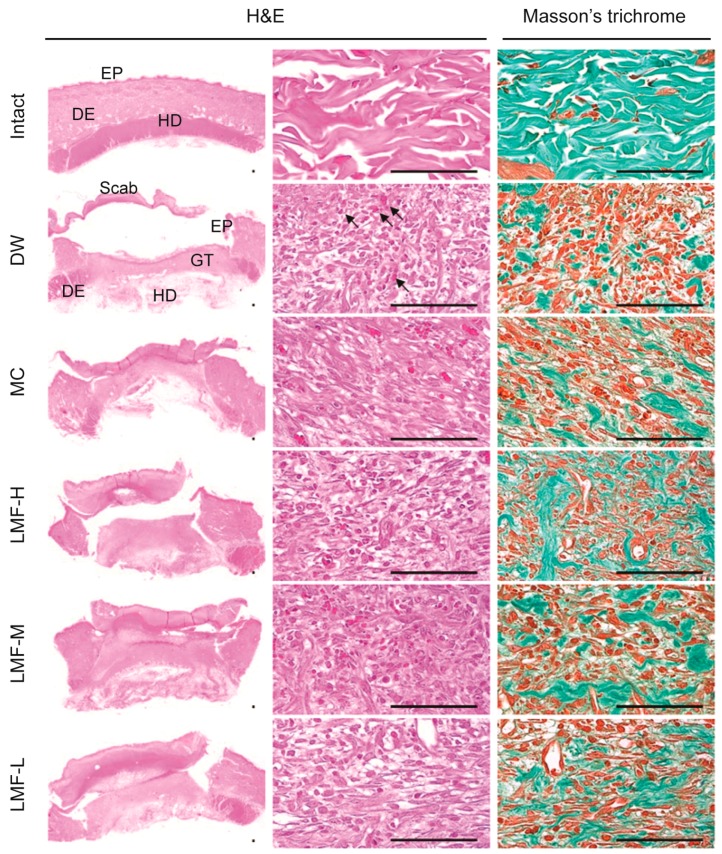
Histopathological changes in granulation tissue on day 4 post-treatment. Median sections from tissue samples of dermal wounds or the corresponding intact dermis were stained with hematoxylin and eosin (H&E) or Masson’s trichrome. Arrows indicates neovascularization. Scale bars = 160 μm. DE = dermis, EP = epithelium, GT = granulation tissue area, and HD = hypodermis.

The LMF groups showed a faster CT_50_ than the DW or MC, therefore, the histopathological changes were examined on days 4 and 7 post-treatment. In hematoxylin and eosin (H&E) stain, a distinct dermal layer, co-organized with basal layer, was observed in the Intact group (non-wounded control), but severe loss of the epithelial layer, with lots of cells showing atrophied and condensed forms, was observed in the DW group (Figure 3). However, the damage to the epithelium was milder in the treatment groups of LMF and MC, accompanied by increased granular tissue and decreased in the infiltration of inflammatory (IF) cells. In addition, increased collagen tissue was observed in the treatment groups compared with the DW group in Masson’s trichrome stain. 

The histomorphometric analyses showed significant main effects of group (*F* = 11.8; *p* < 0.01) for length of the defected epithelium (Table 1). The post-hoc tests versus DW revealed decreases in the length of the treatment groups of LMF and MC (*p* < 0.01). The re-epithelialization was 46.5% ± 5.9%, 41.0% ± 7.7%, 35.7% ± 6.8% and 42.6% ± 9.4% in the LMF-H, LMF-M, LMF-L and MC groups, respectively, while it was 26.4 ± 5.3% in the DW group. There were also significant main effects of group for the areas of granular tissue (*F* = 10.1; *p* < 0.01). The post-hoc test versus DW revealed increases in the granular tissue areas of the LMF groups (*p* < 0.01), but not in that of the MC group. In the granular tissue, the formation of microvessels and collagen deposition were also increased in the groups of LMF and MC compared with the DW group (*p* < 0.05). However, the IF cells were decreased only in the three LMF groups (*p* < 0.05).

### 2.3. Wound Healing Effects on Day 7 Post-Treatment

**Figure 4:**
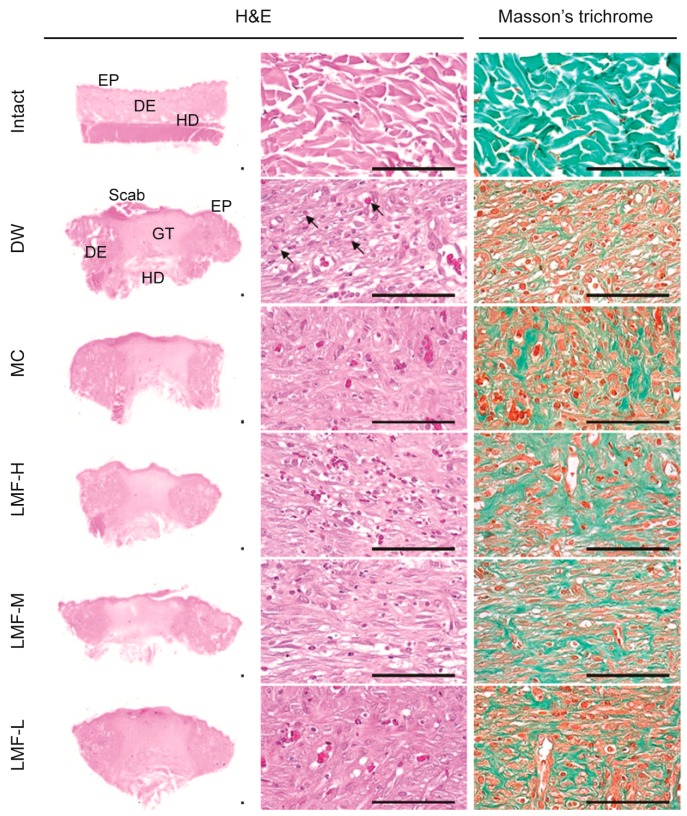
Histopathological changes in granulation tissue on day 7 post-treatment. Median sections from tissue samples of dermal wounds or the corresponding intact dermis were stained with hematoxylin and eosin (H&E) or Masson’s trichrome. Arrows indicate neovascularization. Scale bars = 160 μm. DE = dermis, EP = epithelium, GT = granulation tissue area, and HD = hypodermis.

Similarly, the defected epithelium was observed evidently reduced in the treatment groups of LMF and MC compared with the DW group (Figure 4). The formation of granular tissue was reduced in the treatment groups compared with the DW group, in contrast to the results on day 4 post-treatment, but the formation of microvessels and collagen deposition were still observed increased in the treatment groups. 

The histomorphometric analyses revealed significant decreases in the length of the defected epithelium in the LMF and MC groups compared with the DW group (*F* = 56.5; *p* < 0.01) (Table 2). The re-epithelialization was reached to 94.5% ± 3.4%, 84.9% ± 4.2%, 77.6% ± 5.5% and 75.7% ± 3.9% in the LMF-H, LMF-M, LMF-L and MC groups, respectively, while it was 62.8% ± 6.9% in DW. The ratio was significantly higher in the LMF and MC groups, compared with that in the DW group, and was especially higher in the LMF-H and LMF-M groups, compared with that in the MC group (*p* < 0.01). The area of granular tissue was reduced significantly in the treatment groups of LMF and MC compared with that in the DW (*F* = 19.6; *p* < 0.01). The granular tissue was detected more microvessels and collagen deposition and fewer IF cells in the treatment groups compared with those in the DW (*p* < 0.05), although the values were still different to those in the normal conditions of the Intact group (*p* < 0.05). 

### 2.4. Effects on the Expression of Growth Factors in Dermal Wounds

**Figure 5:**
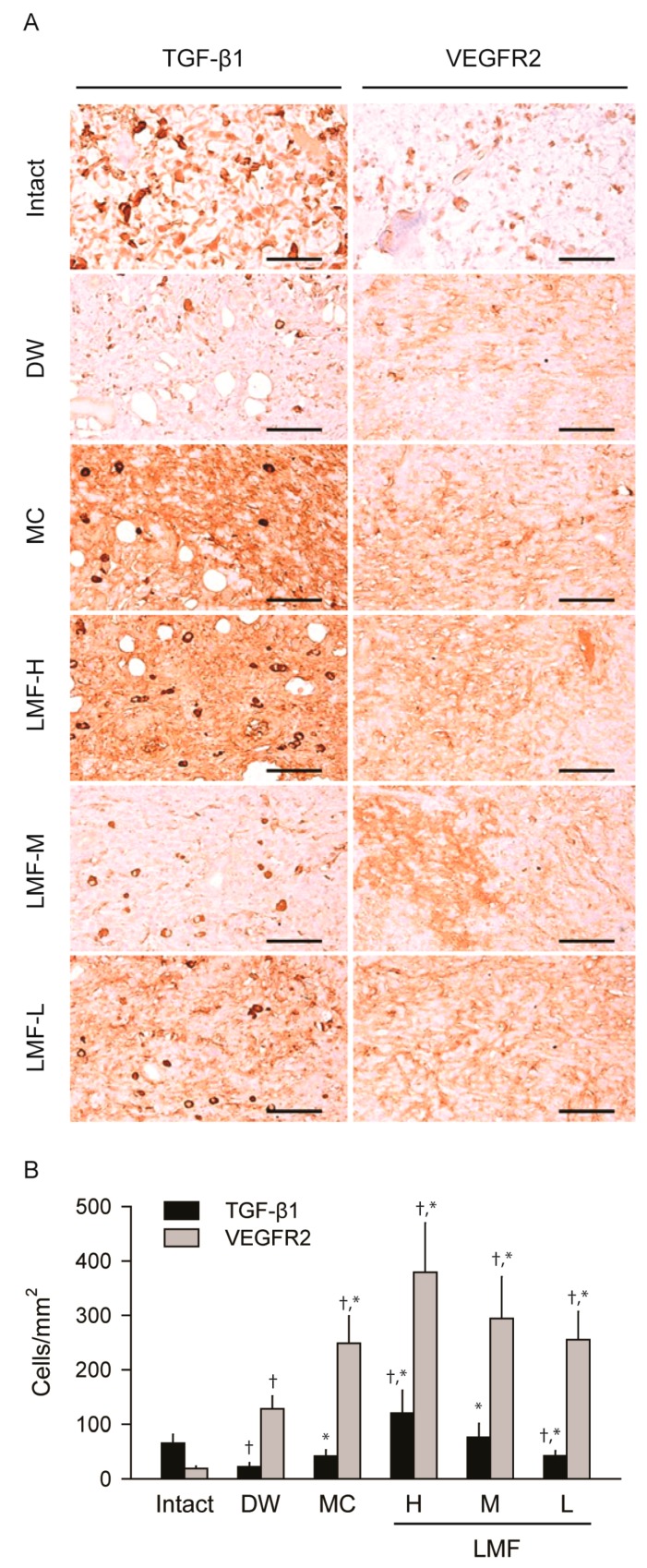
Changes in expression of growth factors on day 4 post-treatment. The serial sections used in Figure 3 were immunostained for transforming growth factor (TGF)-β1 or vascular endothelial growth factor receptor (VEGFR) 2, and then counterstained with hematoxylin (**A**). Scale bars = 160 μm. The numbers of immunostained cells were expressed as means ± SD of 10 wounds (**B**). ^†^ *p* < 0.05 versus the Intact group and * *p* < 0.05 versus the distilled water treated group (DW).

On day 4 post-treatment, the DW group showed an obvious reduction in the number of transforming growth factor (TGF-β1) immunoreactive cells, but increases in vascular endothelial growth factor receptor (VEGFR) 2 immunoreactive cells, compared with the Intact group (Figure 5A). There were significant main effects of group for the immunoreactive cells (*p* < 0.01) (Figure 5B). There were fewer TGF-β1 immunoreactive cells in the DW and LMF-L groups than the Intact, whereas their number was increased only in the LMF-H. When compared with the DW group, the cells were significantly increased in the treatment groups of LMF and MC (*p* < 0.05). The number of VEGFR2 positive cells were increased in the treatment groups compared with those in the Intact or DW group (*p* < 0.05). 

**Figure 6:**
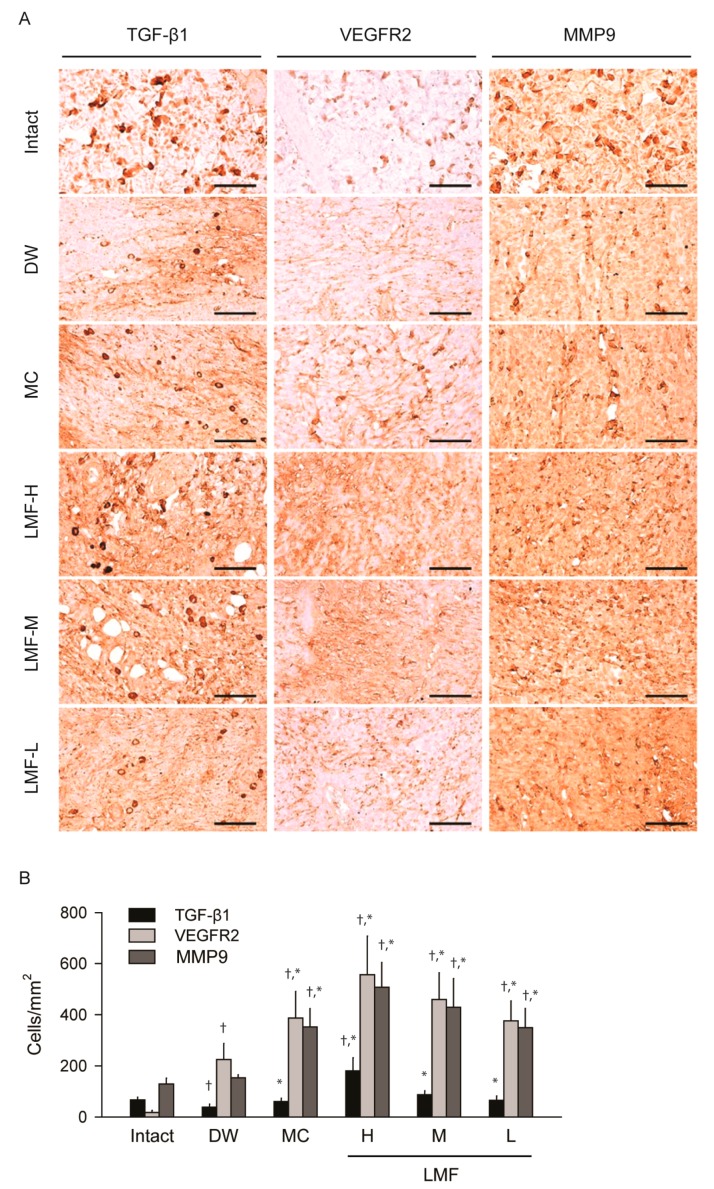
Changes on expression of growth factors on day 7 post-treatment. The other serial sections used in Figure 4 were immunostained for transforming growth factor (TGF)-β1, vascular endothelial growth factor receptor (VEGFR) 2, or matrix metalloproteinases (MMP) 9 and then counterstained with hematoxylin (**A**). Scale bars = 160 μm. The immunostained cells were expressed as means ± SD of 10 wounds (**B**). ^†^ *p* < 0.05 versus the Intact group and * *p* < 0.05 versus the distilled water treated group (DW).

On day 7 post-treatment, the TGF-β1 and VEGFR2 immunoreactive cells were still observed increased in the treatment groups compared with those in the DW (Figure 6A). The immunoreactive cells for matrix metalloproteinases (MMP) 9, as a marker for epithelium remodeling, were increased in the treatment groups compared with those in the Intact or DW. The nonparametric analyses for the TGF-β1-, VEGFR2-, and MMP9-immunoreactive cells showed significant main effects for group (*p* < 0.01) (Figure 6B). Compared with the Intact, the DW group showed significant decreases in the number of TGF-β1 immunoreactive cells and increases in the VEGFR2 immunoreactive cells (*p* < 0.05). There were no differences in the number of MMP9 immunostained cells between the Intact and DW groups. However, the immunoreactive cells for TGF-β1, VEGFR2 and MMP9 were increased significantly in the groups of LMF and MC compared with the DW (*p* < 0.05). 

### 2.5. Effects on Antioxidant Activities in Dermal Wounds

The levels of indices for antioxidant activities showed similar tendencies between the days 4 and 7 post-treatment (Table 3). Nonparametric analyses of the results showed significant differences for group on both days (*p* < 0.01). Compared with the Intact, the DW group showed significant increases in the levels of malondialdehyde (MDA), and decreases in the levels of glutathione (GSH) and the activities of superoxide dismutase (SOD) and catalase (CAT), on days 4 and 7 post-treatment (*p* < 0.05). However, compared with the DW, the MDA levels were decreased significantly in the LMF-H, LMF-M and MC groups on both days (*p* < 0.05), and the levels in the LMF-H and LMF-M groups were not significantly different to those in the Intact on day 7 post-treatment. In addition, the levels of GSH and the activities of SOD and CAT were increased in the treatment groups of LMF and MC compared with those in the DW on both days (*p* < 0.05), excepting for the CAT activity in the LMF-L on day 4 post-treatment. In particular, compared with the Intact, the activities of antioxidant enzymes, CAT and SOD, were similar or even higher in the LMF-H group on days 4 and 7 post-treatment (*p* < 0.05).

## 3. Discussion

Compositions of fucoidans consisting of sulfate, monosaccharide and sugar residue influence anti-inflammatory and antioxidant effects [13,30,31], and the others consisting of galactose and proteins can provide nutrition needed for wound healing [1]. Thus, the LMF extracted from *Undaria pinnatifida*, with substantial fucose and sulfate content, is expected to be a wound-healing accelerator. There have been a few studies on dermal wound healing using fucoidans or LMF extracted from other algal species: one study on the mechanism of fucoidan in in vitro wound healing [32] and two on the efficacy of wound dressing materials containing fucoidan [33,34]. To the best of our knowledge, there are no reports on dermal wound healing using LMF from *Undaria pinnatifida*. The results of the present study demonstrated promising wound-healing effects of LMF, especially LMF-M and LMF-H, in a dermal wound model that enable to compare materials in the same animal [35]. 

Ideally, wound management should achieve rapid wound closure with functional tissue and minimal scarring. The restoration of the tensile strength is achieved by a series of healing processes involving fibroplasia, angiogenesis and the migration of fibroblasts, endothelial cells, and epithelial cells [2,36]. Herein, compared with DW and MC, the topical application of LMF had significantly better effects on wound contraction and produced a faster CT_50_, in a dose-dependent manner. In the LMF-treated wounds, obviously reduced numbers of IF cells were observed on days four and seven post-treatment, but the granular tissue was increased on day four and reduced on day seven. This indicated accelerated wound healing via the anti-inflammatory activities of LMF or promotion of the granulation phase, in agreements of another study [14]. Fucoidans mediate anti-inflammatory effects via reduction of neutrophil adhesion and leukocyte recruitment, or inhibition of proinflammatory cytokines release [19,37]. This might be an intrinsic property of fucoidans, since fucoidans obtained from nine species of brown algae showed similar effects, regardless of their fucose and sulfate contents or other structural features of their polysaccharide backbones [37]. Although it remains unclear how different types of cells with LMF communicate to integrate inflammation and wound healing, this study showed that the LMF treatment accelerates angiogenesis and collagen deposition in the increased granular tissue, resulting in enhanced re-epithelialization. Generally, new microvessel formation contributes to the synthesis of collagen and other proteins via interactions of cells in the dermis and epidermis, in conjunction with the activities of chemical mediators released from inflammatory cells, fibroblasts and keratinocytes [25,38]. It is possible that collagen deposition and the formation of tight cross-links to other collagen molecules and protein would enhance wound contraction, and the proliferation of the various cells comprising the granular tissue would provide a basis for extracellular matrix formation and subsequent re-epithelialization [39]. 

Many substances, including growth factors and cytokines from blood plasma or exudate in the wound, enhance cell proliferation, migration and angiogenesis [2]. LMF treatment increased the number of cells immunoreactive for TGF-β and VEGFR2, which appears to mediate almost all of known cellular responses to VEGF. In particular, LMF-H increased the TGF-β immunoreactive cells to a higher level than that in the Intact group. TGF-β and VEGF are involved in the improvement of wound repair via increases in fibroblast repopulation and angiogenesis [20,25,32]. Fucoidans are known to enhance bioavailability of heparin-binding cytokines, such as bFGF, VEGF and stromal-derived factor-1 (SDF-1), by protecting them from proteolysis, and the increased cytokines promote angiogenesis or granular tissue formation [40,41]. In addition, MMPs, especially MMP2 and MMP9, play key roles as mediators of the collagen matrix during remodeling and re-epithelialization of wound [42,43]. MMP9 has been reported to be increased in serum in response to HMF [44], but not to LMF in a hind limb ischemia rat model [25]. However, in the present study, LMF caused dose-dependent increases in the MMP 9-immunoreactive cells on day 7 post-treatment. This suggested that LMF treatment alters the temporal expression of MMP 9 to accelerate tissue remodeling in response to the increased secretion of various cytokines or their protection from the proteolytic degradation [21]. LMF might be able to modulate growth factor-dependent pathways in the wound-healing process.

Similarly to a previous study [27], LMF treatment reduced lipid peroxidation (MDA) and enhanced the antioxidant defense system, consisting of antioxidant enzymes of SOD and CAT together with the endogenous antioxidant, GSH. Many studies have shown the possibility of using fucoidans as an excellent natural antioxidant to prevent free radical-mediated disease [14,15,31,45], and the effects appear to be related to the sulfate contents [19,46]. This suggests that the substantial sulfate groups of LMF might contribute to strong antioxidant activity. Indeed, similar or lower MDA levels were detected in the LMF-H or LMF-M groups compared with that in the Intact group, representing the normal skin condition, and activation of the antioxidant defense system was increased. Although the antioxidant-related mechanisms are unclear, the complex and coordinated anti-oxidation system might be involved in dermal wound healing. For example, inhibiting nitric oxide (NO) produces anti-inflammatory responses during treatment with fucoidan extracted from *Laminaria japonica* [31], probably because nitric oxide (NO) is an important inflammatory mediator [10]. In turn, the inhibitory effects of LMF on the expression of proinflammatory cytokines such as TNF-α, IL-6, and LI-1β, reduced the proinflammatory enzymes iNOS and COX-2, resulting in decreased oxidative stress [47]. In addition, the interaction of LMF with TGF- β has been reported to be involved in the suppression of oxidative stress [48]. Finally, the reduction of oxidative stress during LMF treatment might be related to the reduced oxidative damages to tissues and cell structures, and the promotion of fibroblast proliferation, angiogenesis and the wound-healing process [49]. These suggest that the antioxidant effects of LMF could be a pivotal regulator of dermal wound healing [50]. 

Fucoidan is commercially available as a health supplement in the USA, UK and Japan. Furthermore, because fucoidans have the potential to enhance dermal fibroblast proliferation and collagen deposition [51], their application as cosmeceuticals has also been studied [13]. In that study, the low molecular weight of LMF was expected to increase its absorption rate and subsequently enhance its beneficial effects, although its sulfate content and molecular weight also influence its biological effects. The beneficial effects of LMF, even at the lowest dose, were similar or better than those of MC, a commercial reference. Considering that factors forwarding to chronic wounds, such as diabetes, pressure ulcers and venous stasis, are increasing as the aging population ages, our results suggest the possibility of using LMF as a topical pharmaceutical agent to promote wound healing in humans.

## 4. Materials and Methods

### 4.1. Reagents

LMF was kindly provided by Bion Co., Ltd. (Daegu, Korea). Briefly, HMF was extracted from the sporophylls of *Undaria pinnatifida*, and then acid-hydrolyzed to produce LMF. The LMF used was the same as that used in our previous studies on its efficacy and toxicity [26,27]. LMF had and average molecular mass of 5 ± 0.6 kDa (polydispersity index = 2.1), and included specific ingredients such as fucose (43.1% *w*/*w*), galactose (12.9% *w*/*w*), uronic acid (2.4% *w*/*w*), sulfate (28% *w*/*w*), protein (5.4% *w*/*w*), moisture (3.2% *w*/*w*) and ash (5% *w*/*w*). Fourier transform infrared spectroscopy analysis showed that the LMF ingredients were similar to those of HMF (Nicolet ECO-RS; Thermo Fisher Scientific, Waltham, MA, USA). The commercial ointment formula, Madecassol Care™ (Dongkook Pharmaceutical Co., Seoul, Korea) containing 1% *Centella asiatica* was used as a reference material [29].

### 4.2. Animals

All animal experiments were performed in accordance with the Guidelines for the Care and Use of Laboratory Animals of Daegu Haany University (Gyeongsan, Korea) (Approval No.: DHU2015-003). Six week-old male Sprague-Dawley rats were purchased from Japan SLC Inc. (Shizuoka, Japan), and allocated randomly at two per polycarbonate cage. They were maintained at 20–25 °C and 40%–45% humidity, under a light:dark cycle of 12 h:12 h. Feed and water were supplied *ad libitum*.

### 4.3. Dermal Wound Induction and Treatment

After 2-week acclimatization, the rats were anesthetized with Zoletil 50^®^ (50 mg/kg, i.p.; Vibac Laboratories, Carros, France), and their dorsal back hair was shaved. Five round wounds per rat were made bilaterally—two wounds on the left side of the dorsal back and three on the right—using an 8 mm-diameter skin biopsy punch (Miltex Inc., York, PA, USA). The wounds were placed at 1.5 cm relative to the back-midline, with cranio-caudal intervals of 2.5 cm. Next day, the wound model animals were regrouped according to similar wound sizes in each of the five regions. Then, in each rat, all five wounds were treated topically with distilled water (DW), Madecassol Care™ (MC) or LMF at 200, 100, and 50 mg/mL (LMF-H, LMF-M and LMF-L, respectively), twice a day. The sites of treatments were rotated sequentially for 100 wounds (*n* = 20 rats) (Figure 1). The DW and LMF treatment was applied in a volume of 200 μL as a liquid, and MC ointment was in an amount that slightly covered the wound. The rat was euthanized with CO_2_ gas on days 4 and 7 post-treatment, and samples for the wound defects and the corresponding intact dermis were collected for biochemical or histopathological analyses.

### 4.4. Assessment of the Wound Area

The wound area was traced on a transparent film daily after wounding and the area was analyzed using a computer-based automated image analyzer (*i*Solution FL ver. 9.1, IMT *i*-solution Inc., Quebec, QC, Canada). It was expressed as kinetic curve with contraction ratios (%) as following equation:

Wound contraction ratio (%) = (*A*_0_ − *A*_t_)/*A*_0_ × 100

where, *A*_0_ and *A*_t_ are the initial wound area and the area at time t, respectively.

The half-closure time (CT_50_) was estimated using linear regression on the curve.

### 4.5. Biochemical Analyses for Antioxidant Activities

A portion of dermal samples was homogenized as 10% in cold phosphate buffered saline (PBS, pH 7.2), and it was then centrifuged at 12,500× *g* for 60 min at 4 °C [10]. The supernatants were subjected to biochemical assays or stored at −80 °C until use. For lipid peroxidation, the MDA content was determined using the thiobarbituric acid reaction. The homogenates were mixed with 20% trichloroacetic acid (Merck, San Francisco, CA, USA), and centrifuged at 12,000× *g* for 20 min. The supernatant was added to thiobarbituric acid (Sigma-Aldrich, St. Louis, MO, USA), and then boiled for 20 min. The contents were assessed at 532 nm in a UV/visible spectrometer (OPTIZEN POP, Mecasys, Daejeon, Korea), and expressed as nM/mg protein. For GSH, the homogenates were added by 5% trichloroacetic acid (Merck) and centrifuged at 12,000× *g* for 40 min at 4 °C. The supernatants were mixed with 0.2 M phosphate buffer (pH 8.0) and 0.6 mM 2-nitrobenzoic acid (Sigma-Aldrich). The contents were assessed at 412 nm and expressed as μg/mg protein. For superoxide dismutase (SOD), the homogenates were mixed with 0.6 mM EDTA (Sigma-Aldrich), 0.25 M sodium carbonate (Merck) and 3.0 mM epinephrine (Sigma-Aldrich). The contents were assessed at 470 nm, and expressed as U/mg protein. For CAT, the homogenates were mixed with 0.05 M phosphate buffer (pH 7.0), 5.0 mM hydrogen peroxide (Merck) and 0.1 mL of diluted enzyme (skin homogenate). The CAT activity was assessed at 240 nm at 30 s intervals for 3 min, and expressed as U/mg protein. The activity was defined as the amount of enzyme required to decompose 1 nM of hydrogen peroxide per min. Protein assays were performed using bovine serum albumin as a standard (Invitrogen, Carlsbad, CA, USA).

### 4.6. Histopathology

Another portion of dermal samples was fixed in 10% neutral buffered formalin, and cross-trimmed to leave the central wound regions. The samples were paraffin-embedded, and serially sectioned at a thickness of 3 μm. The sections were stained with H&E or Masson’s trichrome for collagen fibers [52,53], and examined under a light microscope (E400, Nikon, Tokyo, Japan). The histomorphometric analyses were performed for length of the desquamated epithelium (mm), numbers of microvessels (vessels/mm^2^) and infiltrated inflammatory (IF) cells (cells/mm^2^), collagen depositions (%/mm^2^) and granulation tissue areas (mm^2^), using a computer-based automated image analyzer as described previously [52,53]. The histopathologist was blinded to the treatment group. Re-epithelization of the central wound was calculated as the following formula [53]:

Re-epithelization (%) = (*L*_0_ − *L*_t_)/*L*_0_ × 100

where, *L*_0_ and *L*_t_ are the initial wound diameter (8 mm) and the length at time t, respectively.

### 4.7. Immunohistochemistry

The serial sections were immunostained for TGF-β1, VEGFR2 or MMP9. After antigen retrieval in 10 mM citrate buffers (pH 6.0), the section was treated with 3% H_2_O_2_ in methanol for 30 min, and blocked with normal horse serum for 1 h. It was then incubated overnight at 4°C in humidity chamber with rabbit polyclonal antibodies (Abcam, Cambridge, UK); anti-TGF-β1 (ab92486, dilution 1:100), VEGFR-2 (ab45010, dilution 1:100) or MMP-9 (ab38898, dilution 1:100). The sections were incubated with biotinylated universal secondary antibody and ABC reagents (Vector Labs, Burlingame, CA, USA) for 1 h, and the immunoreactivity was visualized using a peroxidase substrate kit. The sections were rinsed in 0.01 M PBS three-times between each step. The cells occupying over 20% of immunoreactivities were regarded as positive, and the number of immunoreactive cells was measured using automated image analyzer (cells/mm^2^) based on a previous report [54].

### 4.8. Statistical Analyses

All data are expressed as means ± SD for 10 samples per group. Initially, the data were analyzed by the Levene test for homogeneity of variance. If the test indicated no significances, the data were examined by analysis of variance (ANOVA), followed by a least significant difference post-hoc test. However, if there were significances, a Kruskal-Wallis H test was conducted for nonparametric comparisons, followed by a Mann-Whitney U post-hoc test. The post-hoc test was further examined by Bonferroni correction. The kinetics of wound contraction was examined by a 3-way ANOVA with main factors of group and wound regions, and day was treated as repeated measurements. The analyses focused on the differences among treatment groups. A *p*-value < 0.05 was considered statistically significant.

## Figures and Tables

**Table 1:** Histomorphometric analyses on full-thickness dermal wounds on day 4 post-treatment.

Group	Defected Epithelium (mm)	Granulation Tissue of Dermal Wound or Intact Dermis			
		Area (mm^2^)	Microvessels (n/mm^2^)	IF Cells (n/mm^2^)	Collagen Tissue (mm^2^)
Intact	NA	NA	4.30 ± 2.31	7.80 ± 2.78	65.01 ± 11.20
DW	5.89 ± 0.42	4.15 ± 0.13	56.30 ± 18.82 ^†^	291.10 ± 135.79 ^†^	21.76 ± 6.79 ^†^
MC	4.59 ± 0.75 *	4.53 ± 0.32	159.10 ± 39.40 ^†,^*	176.20 ± 30.31 ^†^	37.72 ± 11.03 ^†,^*
LMF-H	4.28 ± 0.47 *	5.37 ± 0.62 *	228.30 ± 39.65 ^†,^*	84.80 ± 11.46 ^†,^*	43.68 ± 10.22 ^†,^*
LMF-M	4.28 ± 0.47 *	5.20 ± 0.68 *	169.70 ± 21.02 ^†,^*	130.30 ± 27.42 ^†,^*	40.45 ± 10.38 ^†,^*
LMF-L	5.14 ± 0.54 *	5.08 ± 0.55 *	134.70 ± 25.18 ^†,^*	171.70 ± 17.58 ^†,^*	37.93 ± 10.54 ^†,^*

Histomorphometric analyses were performed in hematoxylin and eosin (H&E) and Masson’s trichrome stains of median sections from tissue samples of dermal wounds or the corresponding intact dermis. Values are expressed as means ± SD of 10 wounds. IF cells = infiltrated inflammatory cells composed of neutrophil or mononuclear cells. NA = not associated. ^†^ *p* < 0.05 versus the Intact group and * *p* < 0.05 versus the distilled water treated group (DW).

**Table 2:** Histomorphometric analyses on full-thickness dermal wounds on day 7 post-treatment.

Group	Defected Epithelium (mm)	Granulation Tissue of Dermal Wound or Intact Dermis			
		Area (mm^2^)	Microvessels (n/mm^2^)	IF Cells (n/mm^2^)	Collagen Tissue (mm^2^)
Intact	NA	NA	6.40 ± .95	7.90 ± 3.07	68.27 ± 10.24
DW	2.98 ± 0.55	5.77 ± 1.21	135.90 ± 23.84 ^†^	151.80 ± 46.24 ^†^	32.64 ± 10.36 ^†^
MC	1.94 ± 0.31 *	3.76 ± 1.27 *	187.40 ± 19.80 ^†,^*	85.50 ± 29.52 ^†,^*	45.25 ± 4.39 ^†,^*
LMF-H	0.44 ± 0.27 *	2.32 ± 0.43 *	225.60 ± 19.39 ^†,^*	27.00 ± 10.83 ^†,^*	55.45 ± 11.00 ^†,^*
LMF-M	1.21 ± 0.33 *	2.56 ± 0.81 *	209.30 ± 29.36 ^†,^*	49.10 ± 12.21 ^†,^*	51.16 ± 11.80 ^†,^*
LMF-L	1.79 ± 0.44 *	3.82 ± 0.94 *	187.20 ± 23.30 ^†,^*	87.40 ± 32.53 ^†,^*	45.06 ± 7.51 ^†,^*

Histomorphometric analyses were performed in hematoxylin and eosin (H&E) and Masson’s trichrome stains of median sections from tissue samples of dermal wounds or the corresponding intact dermis. Values are expressed as means ± SD of 10 wounds. IF cells = infiltrated inflammatory cells composed of neutrophil or mononuclear cells. NA = not associated. ^†^ *p* < 0.05 versus the Intact group and * *p* < 0.05 versus the distilled water treated group (DW).

**Table 3:** Antioxidant activities in full-thickness dermal wounds.

Group	MDA (nM/mg Protein)	GSH (μg/mg Protein)	SOD (U/mg Protein)	CAT (U/mg Protein)
*Day 4*				
Intact	2.35 ± 0.55	2.61 ± 0.49	1.89 ± 0.25	6.75 ± 0.93
DW	4.36 ± 0.65 ^†^	1.06 ± 0.23 ^†^	1.05 ± 0.18 ^†^	4.61 ± 0.67 ^†^
MC	3.13 ± 0.34 ^†,^*	1.88 ± 0.21 ^†,^*	1.54 ± 0.20 ^†,^*	5.43 ± 0.41 ^†^
LMF-H	2.91 ± 0.20 *	2.17 ± 0.28 ^†,^*	1.77 ± 0.15 *	5.85 ± 0.64 *
LMF-M	3.10 ± 0.25 ^†,^*	1.90 ± 0.37 ^†,^*	1.53 ± 0.13 ^†,^*	5.60 ± 0.44 *
LMF-L	3.40 ± 0.43 ^†^	1.69 ± 0.29 ^†,^*	1.40 ± 0.26 ^†,^*	5.26 ± 0.20 ^†^
*Day 7*				
Intact	2.38 ± 0.52	2.67 ± 0.43	1.84 ± 0.24	6.89 ± 0.79
DW	4.88 ± 1.23 ^†^	1.17 ± 0.27 ^†^	1.11 ± 0.17 ^†^	4.89 ± 0.36 ^†^
MC	3.40 ± 0.50 ^†,^*	2.16 ± 0.21 *	1.63 ± 0.16 ^†,^*	5.71 ± 0.64 ^†,^*
LMF-H	2.89 ± 0.48 *	2.51 ± 0.30 *	2.02 ± 0.17 ^†,^*	6.75 ± 0.64 *
LMF-M	3.10 ± 0.29 *	2.28 ± 0.21 *	1.78 ± 0.15 *	6.27 ± 0.40 ^†,^*
LMF-L	3.37 ± 0.45 ^†^	2.17 ± 0.22 *	1.65 ± 0.24 ^†,^*	5.84 ± 0.57 ^†,^*

The granulation tissue from the dermal wounds or the corresponding intact dermis were assessed for contents of malondialdehyde (MDA) and glutathione (GSH) and enzyme activities of superoxide dismutase (SOD) and catalase (CAT), on days 4 and 7 post-treatment. Values are expressed as means ± SD of 10 wounds. ^†^ *p* < 0.05 versus the Intact group and * *p* < 0.05 versus the distilled water treated group (DW).
